# Remodeling of mitochondrial morphology and function: an emerging hallmark of cellular reprogramming

**DOI:** 10.15698/cst2019.06.189

**Published:** 2019-05-27

**Authors:** Anuj Rastogi, Piyush Joshi, Ela Contreras, Vivian Gama

**Affiliations:** 1 Department of Cell & Developmental Biology, Vanderbilt University, Nashville, TN 37240.; 2 Neuroscience Program, Vanderbilt University Medical Center, Nashville, TN 37240.; 3 Vanderbilt Brain Institute, Vanderbilt University Medical Center, Nashville, TN 37240.; 4 Vanderbilt Center for Stem Cell Biology, Vanderbilt University, Nashville, TN 37240.

**Keywords:** BCL-2 family, mitochondria, mitochondrial dynamics, apoptosis

## Abstract

Research in the stem cell field has traditionally focused on understanding key transcriptional factors that provide pluripotent cell identity. However, much less is known about other critical non-transcriptional signaling networks that govern stem cell identity. Although we continue to gain critical insights into the mechanisms underlying mitochondrial morphology and function during cellular reprogramming – the process of reverting the fate of a differentiated cell into a stem cell, many uncertainties remain. Recent studies suggest an emerging landscape in which mitochondrial morphology and function have an active role in maintaining and regulating changes in cell identity. In this review, we will focus on these emerging concepts as crucial modulators of cellular reprogramming. Recognition of the widespread applicability of these concepts will increase our understanding of the mitochondrial mechanisms involved in cell identity, cell fate and disease.

## INTRODUCTION

A paradigm shift from considering mitochondria mere powerhouses of the cell to realizing that they are a major regulatory hub began two decades ago with two discoveries: (1) that cytochrome *c*, a protein known previously only as an electron transporter, triggers cell disassembly during apoptosis once released into the cytoplasm [1]; and (2) that the B-Cell Lymphoma-2 (BCL-2) family controls this release [2]. Another shift followed from discovering that mitochondria are highly dynamic organelles, which fuse into each other and divide rapidly and reversibly in response to internal and external environmental cues. Subsequent discoveries established that mitochondrial fusion and fission (division), which are collectively known as mitochondrial dynamics [3, 4], are involved in aging [5], neurodegenerative diseases [6], and tumorigenesis [7–9]. Recent studies provide compelling evidence for a novel function of mitochondrial dynamics in human pluripotent stem cells (hPSCs). These investigations have revealed that the BCL-2 family is essential in hPSCs not only during cell death, but also through its ability to regulate the mitochondrial network, which is key for other cell fate decisions, such as self-renewal and pluripotency [10–12].

Stem cells can be divided into three main types: embryonic stem cells (ESCs), adult stem cells, and induced pluripotent stem cells (iPSCs). ESCs and iPSCs are defined by two key properties: self-renewal (defined as the ability to proliferate without lineage commitment) and pluripotency (defined as the ability to differentiate into the three main tissue lineages). Since the first human ESC (hESC) line was derived in 1998, hESCs have become efficient tools to study processes of human development and various aspects of human diseases, like cancer [13]. Since cells from the inner cell mass, from which ESCs are derived, can give rise to all tissues derived from germ layers (endoderm, mesoderm, and ectoderm), genomic instability is especially risky for their self-renewal and organ specific differentiation. Thus, not surprisingly, these cells are endowed with exquisite mechanisms to respond rapidly to apoptosis-inducing stress. Various studies have provided evidence of changes in apoptosis regulation, mitochondrial dynamics and metabolic function and regulation during the process of reprogramming (i.e. reverting a differentiated cell into a stem-like state, through the expression of master pluripotency transcription factors such as, OCT4 (Octamer-binding transcription factor 4), SOX2 (SRY(sex-determining region Y)-box 2), KLF4 (Kruppel-like factor 4) and *c-*MYC, collectively known as OSKM) [14, 15].

**Figure 1:**
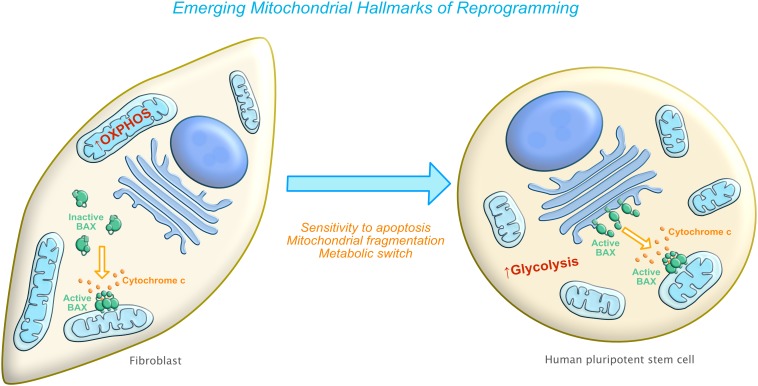
FIGURE 1: Emerging mitochondria-related hallmarks of reprogramming. Left side: Differentiated cells (such as fibroblasts) have an elongated mitochondrial network with a preference for oxidative phosphorylation (OXPHOS) as a source of energy and are relatively resistant to apoptosis stimuli. Right side: After reprogramming, cells now have a fragmented mitochondrial network with a switch to using glycolysis as a preferred bioenergetic source. Cells become increasingly sensitive to cell death due to at least two main mechanisms: increased mitochondrial priming and pre-activated BAX.

Mitochondria-related hallmarks of reprogramming are beginning to emerge. In this review, we specifically discuss three biological capabilities acquired during the multistep transition of a somatic cell into a pluripotent stem cell. They include: inherent sensitivity to apoptosis, changes in mitochondrial morphology and localization, and a modified functional state of the mitochondria (i.e changes in metabolic requirements) [10, 11, 13–17] (**Figure 1**). In the following sections, we will explore the known underlying mechanisms involved in these changes highlighting recent discoveries as well as describe areas that are open to more detailed investigation. While we propose that these three mitochondria-related hallmarks constitute a requirement for reverting a differentiated cell into a stem cell, there are other mitochondrial events such as mitochondrial biogenesis, mitochondrial trafficking and mitochondrial transcription that also undergo dramatic changes during cellular reprogramming and that could also be essential for successfully reverting cell fate. Additional studies are needed to elucidate the impact of these events on the reprogramming process.

## INCREASED SENSITIVITY TO CELL DEATH

**Figure 2:**
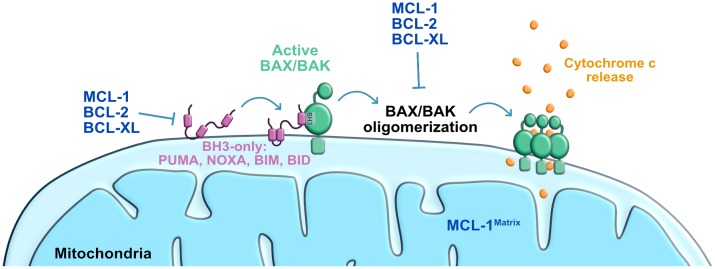
FIGURE 2: Scheme of the mitochondrial pathway of apoptosis. Stress-induced activation of BAX/BAK leading to mitochondrial outer membrane permeabilization and cytochrome c release to the cytosol. Anti-apoptotic proteins block BAK/BAX activation/oligomerization induced by BH3-only proteins. BCL-XL: B-cell lymphoma extra-large; MCL-1: myeloid cell leukemia 1; BID: BH3-interacting domain death agonist; BIM: BCL-2 interacting mediator of cell death; PUMA: P53-upregulated modulator of apoptosis.

The apoptotic process involves morphologic changes including cell constriction, chromatin condensation, nuclear envelope disruption followed by nucleus breakdown to discrete bodies, plasma membrane blebs formation, and finally the break-up of the cell into apoptotic bodies [18]. Activated cysteine proteases known as caspases cleave many vital cellular proteins (nuclear scaffold, cytoskeleton, etc.) followed by nuclear DNA degradation [19, 20]. Caspase dependent apoptosis is classified into extrinsic and intrinsic pathways, mediated by death ligands and mitochondria respectively [21]. Our focus in this review will be on the mitochondrial pathway of apoptosis (**Figure 2**). The extrinsic pathway of apoptosis has been described extensively in several excellent reviews [22–24].

The intrinsic apoptosis pathway, also known as the mitochondria mediated cell death pathway, is a form of regulated cell death initiated by a variety of perturbations including DNA damage [24]. These stresses induce the activation of the transcription factor P53 that then initiates a response to arrest cell cycle and repair DNA [25, 26]. If repair is not achieved, an apoptosis response is triggered leading to the activation of the BCL-2 associated X (BAX) and/or BCL-2 antagonist killer 1 (BAK1; best known as BAK), these pro-apoptotic effector proteins of the BCL-2 family then permeabilize the outer mitochondrial membrane [27–33. BAX and BAK can be directly activated via binding to a subset of BH3-only proteins known as “activators” (i.e. BCL-2 interacting mediator of cell death (BIM), BCL-2 interacting domain death agonist (BID), P53-upregulated modulator of apoptosis (PUMA), and phorbol-12-myristate-13-acetate-induced protein 1 (PMAIP1, best known as Noxa) [32, 33]. The anti-apoptotic members of the BCL-2 family (e.g. BCL-2, apoptosis regulator (BCL-2), BCL2-like 1 (best known as BCL-XL) and Myeloid Leukemia Sequence 1 (MCL-1)) can also bind to BAX and prevent its function. BH3-only proteins known as “sensitizers” (e.g. BCL2 associated agonist of cell death (BAD), and BCL-2 modifying factor (BMF)) inactivate the anti-apoptotic BCL-2 family proteins, and therefore may also be needed for efficient BAX and BAK activation [24, 32–34]. Early studies examining the interaction between BH3-only proteins and the anti-apoptotic proteins revealed differential binding patterns that have been exploited to examine the apoptotic vulnerability of various cells of interest [35–38]. Mitochondrial outer permeabilization induced by BAX and BAK, then results in the release of cytochrome *c*, oligomerization of the Apoptotic Protease Activating Factor-1 (APAF-1) and the activation of the executioners of apoptosis, a family of cysteine proteases termed caspases [39–41]. Caspases cleave intracellular targets [42], resulting in the morphological changes previously described, and cellular demise [24, 43].

**Figure 3:**
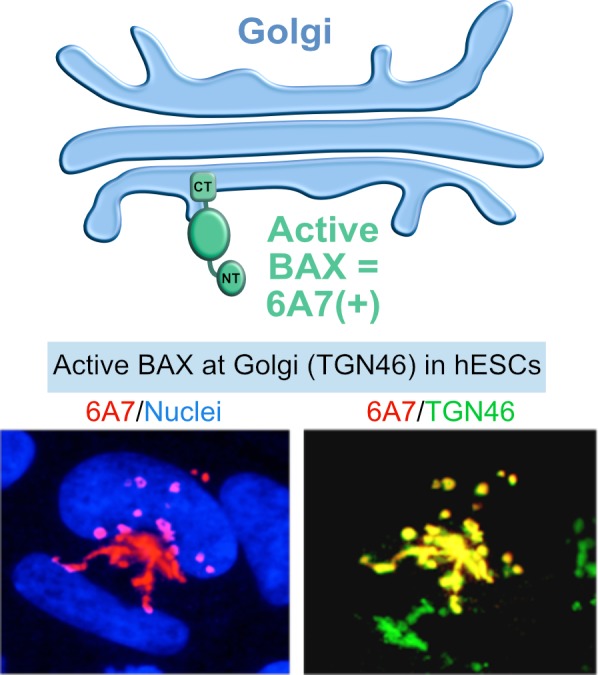
FIGURE 3: BAX activation in stem cells. Besides high mitochondrial priming, human ESCs also have an active form of BAX at the Golgi that rapidly translocates to the mitochondria under apoptosis stimuli. Active form of BAX: BAX 6A7; Golgi apparatus marker: TGN46.

In contrast to differentiated cells, BAX is kept in an active state in hESCs and sequestered at the Golgi network [14, 44] (**Figure 3**). Upon DNA damage, active BAX rapidly translocates from the Golgi to mitochondria, triggering apoptosis. Such a rapid response may be critical for preventing the propagation of aberrant cells in the developing embryo, or the emergence of a cancerous cell in the tissues of an adult organism, acting as sort of “cocked gun”. This “cocked gun” is disarmed once the cell differentiates [14, 44], implying that the cell uses specific mechanisms for inactivation, which are unknown. An additional study followed these observations and determined that mitochondrial priming also contributes to the increased apoptosis sensitivity of hESCs compared to differentiated cells [15]. The balance between pro and anti-apoptotic factors shifts more towards the apoptotic threshold in hESCs [15, 44]. How the high mitochondrial priming state is initiated and maintained in hPSCs, as well as how it is reset early during differentiation is not known. Considering the phenomenon of mitochondrial priming and the fact that BAX is constitutively activated in hPSCs, we hypothesized that proteins of the BCL-2 family have uncharacterized functions in hPSCs that are unrelated to cell death.

Interestingly, both caspases and P53 play a major part in the generation of iPSCs. However, the mechanistic details of their involvement in somatic cell reprogramming are unknown. Apoptosis serves a defensive role by eliminating genetically abnormal and damaged cells from the pluripotent population [45–48]. Thus, antagonizing apoptosis by overexpression of anti-apoptotic BCL-2 or via repression of P53 significantly improves the efficiency of iPSC generation [49, 50]. Previous studies showed that Caspase-8 deficient animals die in their early developmental stages due to improper differentiation of neural precursors [51]. This correlation between caspases and the ability of embryonic stem cells to self-renew or differentiate was later demonstrated by the report that caspase-3 and caspase-9 mutant mouse ESCs (mESCs) are unable to differentiate [52]. A caspase-dependent degradation site in the stem cell factor NANOG is conserved between mice and human protein homologues, providing insight into the mechanism by which caspases regulate reprogramming efficiency [52]. Caspase-3 dependent degradation of NANOG was reported as a key factor leading to the differentiation of hESCs and iPSCs in the absence of basic fibroblast growth factor [53]. In contrast to their role in ESCs, activated caspase-3 and caspase-8 via degradation of Retinoblastoma protein (RB), help in the de-differentiation of human fibroblasts into iPSCs [54]. Thus, caspases play a dual role by driving dedifferentiation of somatic stem cells and differentiation of ESCs, in the context of cellular and substrate availability. The use of caspase inhibitors may work as a fine regulator for differentiating ESCs/iPSCs and for reprogramming iPSCs. Hence, further investigation of underlying molecular mechanisms of caspase regulation is needed.

Numerous studies have demonstrated that P53 is essential for embryonic development and cellular differentiation [55]. P53 transcriptional activity in cellular differentiation is context dependent having contradictory results in various cell types [55]. For instance, P53 enhances the differentiation of both murine and human ESCs in the presence of retinoic acid and DNA damage [56, 57] whereas, P53 is also shown to promote hematopoietic stem cell (HSC) quiescence and inhibit neural stem cell proliferation [58, 59]. Use of the MDM2 inhibitor nutlin-3a results in rapid accumulation of P53 and subsequent induction of apoptosis and rapid differentiation in hESCs, but it has a minimal effect in somatic cells [60, 61]. Similar to the observations that stabilization of P53 plays a role in differentiation of ESCs, various studies have shown that inactivation of P53 helps in reprogramming of somatic cells to iPSCs [49, 62, 63]. Considering that iPSCs and malignant somatic cells share similar characteristics (higher proliferation, transcriptional and metabolic status), the negative role of P53 in cellular reprogramming is not unexpected. Mechanistic insights for P53's inhibitory role in cellular reprogramming involves P53 mediated targeting of miR-34a that suppresses the expression of Sox2, MYC and NANOG [64], regulation of CDKN1A that attenuates cell division [48] and inhibition of specific epithelial genes that further suppress mesenchymal to epithelial transition [46].

Reprogramming efficiency of somatic cells to iPSCs is reduced in the absence of c-MYC. However, the tendency of MYC to cause apoptosis also represents a roadblock to reprogramming. A recent study indicates that reprogramming of mouse embryonic fibroblasts (MEFs) is enhanced in the absence of BAK and BAX under OKSM conditions [65]. These studies demonstrate that mitochondrial apoptosis imposes a strong MYC-dependent block to somatic cell reprogramming [10, 50, 65, 66]. Hence, although MYC expression favors reprogramming by regulating cellular processes (transcription, translation, ATP generation) [67, 68], its tendency to sensitize cells to mitochondrial apoptosis is an interesting paradox. Intriguingly, high γ-H2A.X deposition has been described in hPSCs and it has been linked to the global chromatin de-condensation required by pluripotent stem cells in order to dynamically activate transcriptional programs in response to environmental cues [69]. How these epigenetic changes are linked to increased mitochondrial apoptosis is not known. Understanding the exact mechanisms by which reprogramming induces epigenetic modifications, constitutive apoptosis sensitization and priming of pluripotent stem cells is an area in need of more investigations.

## INCREASED MITOCHONDRIAL FISSION

Mitochondrial movement and fragmentation were first observed almost 100 years ago [70]. For decades these observations remained unexplored and the idea of mitochondria as isolated and static “jelly-bean” structures that serve as “power houses” of the cells became an unrefuted dogma. The development of new technologies made it easier to track mitochondria in live cells. These studies revealed the remarkable ability of mitochondria to move and to continually divide and fuse [71–73]. Further studies have demonstrated that mitochondrial dynamics are crucial for normal physiology from yeast to mammals. Emerging studies demonstrate a connection between mitochondrial dynamics machinery, apoptosis and mitophagy. In addition, several human diseases result from mutations in fusion and fission proteins further highlighting the relevance of mitochondrial fission and fusion events in cellular homeostasis [8, 74–77].

Interestingly, as differentiated cells transition to a stem cell state, the mitochondrial network undergoes dramatic changes. Mitochondria of stem cells contain functionally immature mitochondria with a globular shape, poorly developed cristae, and perinuclear localization, an indicator of a less active mitochondrial state [78, 79], whereas differentiated cells, like fibroblasts, have a complex morphology with well-developed cristae, dense matrix, and elongated appearance. It is not completely understood how increased mitochondrial fragmentation is maintained in stem cells, however, constitutive activation of the dynamin-related guanosine triphosphates (GTPases) is likely a contributing factor [79].

Dynamin-related GTPases control mitochondrial dynamics by allowing opposing processes of division and fusion to work in concert maintaining overall shape and number of mitochondria [76, 79]. Large dynamin-related GTPase proteins (DRPs) are highly conserved and have the ability to self-assemble and hydrolyze GTP to control mitochondrial fusion and fission [80, 81]. In mammals, fission or fragmentation of the mitochondrial network is mediated by dynamin related protein-1 (DRP-1). DRP-1 is primarily localized in the cytosol and recruited to mitochondria during fission. Activation of DRP-1 is mediated by post translational modifications including phosphorylation, sumoylation, O-GlcNAcylation and ubiquitination which are thought to enhance its recruitment to mitochondrial receptors [83]. The impact of these post-translational modifications for maintaining the homeostatic mitochondrial fragmentation in pluripotent stem cells is not known. Modified DRP-1 oligomerizes around mitochondria and constricts the network severing both mitochondrial inner and outer membranes. The structural domains and mechanistic details of action for DRP1 have been clearly elucidated [8, 80–86]. DRP-1 is also involved in promoting mitochondrial fragmentation during apoptosis [87] (reviewed in next section).

Fusion of mitochondria requires Mitofusin 1 (MFN1), Mitofusin 2 (MFN2), and Optic Atrophy 1 (OPA1) to fuse the outer and inner mitochondrial membranes [88–90]. Fusion is key for mitochondrial DNA (mtDNA) homogenization and assembly of electron transport chain. MFN1 and MFN2 are anchored in the outer mitochondrial membrane and allow for formation of homo- or hetero-dimers with MFNs facilitating fusion. The inner membrane is fused afterwards in a similar fashion by OPA1. OPA1 is localized to the inner mitochondrial membrane with GTPase domain exposed to inner membrane space. Overexpression of OPA1 leads to fragmentation of mitochondria, however, effects on cell death are not clear [91]. The mechanisms by which the activity of the dynamin-related proteins involved in fusion is regulated are not well understood, but data from *in vitro* assays illustrates that inner and outer mitochondrial fusion are separable and mechanistically different [92, 93]. Fission is necessary for cell division and for mitophagy when damaged mitochondria have to be segregated. As mentioned previously, mitochondrial dynamics are crucial for normal physiology from yeast to mammals [94]. Imbalance in the process of fusion and fission leads to severe pathophysiological conditions. These range from the inability to survive past mid-gestation in MFN1, MFN2, OPA1, or DRP-1 deficient mice [90, 90, 95–97], to neurodegenerative diseases such as Charcot-Marie-Tooth syndrome and dominant optic atrophy [88, 89, 98, 99] caused by mutations in MFN2 and OPA1.

**Figure 4:**
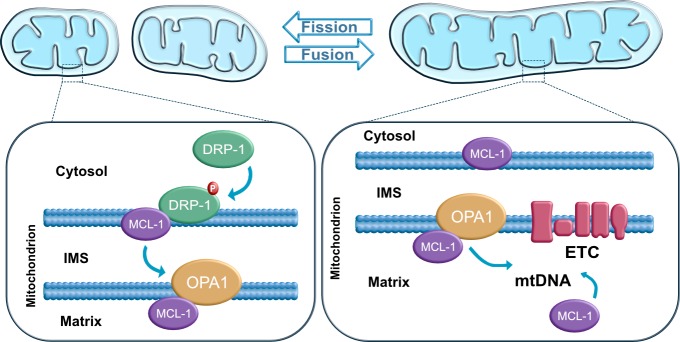
FIGURE 4: Mitochondrial dynamics. Mitochondrial fusion and fission are regulated by guanosine triphosphatases (GTPases) proteins: DRP1 mediates fission, OPA1 and Mitofusins (not shown) regulate mitochondrial fusion. In stem cells, the anti-apoptotic protein MCL1 has been shown to interact with DRP1 at the outer mitochondrial membrane and with OPA1 at the matrix.

The BCL-2 family has recently been implicated as a key factor in maintaining stem cell self-renewal and pluripotency. Inhibition of pro-apoptotic BAX and BAK proteins has been reported to be required for mitochondrial fusion [80, 100–102]. BAX has been suggested to regulate fusion by interacting with MFN1 and/or MFN2 [102, 103]. BCL-xL, an anti-apoptotic protein, has been shown to be highly expressed at the mitochondria of adult neurons and required for normal brain development [104]. BCL-xL appears to affect mitochondrial dynamics in mammalian neurons resulting in an increment of the length/size of mitochondria and the localization of mitochondria to synapses [105, 106]. Furthermore, the anti-apoptotic protein MCL-1 appears to be involved in the regulation of mitochondrial dynamics and the maintenance of pluripotency [10]. MCL-1 appears to interact with DRP-1 and OPA1 in hPSCs, and potentially other BCL-2 family members. This interaction may be critical for the modulation of mitochondrial dynamics (**Figure 4**). A recent study further demonstrates that the BH3-only protein BID also regulates mitochondrial morphology and cristae organization [12]. The functional implication of a potential MCL-1 and BID interaction in maintaining pluripotency and self-renewal ability of hPSCs has not yet been explored. Revealing the mechanistic link between the mitochondrial dynamics machinery and the BCL-2 family represents a unique opportunity for increasing our understanding of how these mitochondrial signaling pathways interact to regulate cell fate.

### Mitochondrial remodeling during apoptosis

The mitochondrial pathway of apoptosis causes the remodeling of mitochondrial structure that ultimately enables the release of cytochrome *c*, the defining moment of apoptosis [87, 101, 107–112]. Some studies suggest that BAX/BAK-mediated outer mitochondrial membrane permeabilization is not sufficient for cytochrome *c* release during apoptosis, but rather it requires mitochondrial fragmentation to occur first. Activation of BAX and BAK may lead to changes in mitochondrial cristae structure mediated by OPA1 monomerization which drives remodeling and opening of cristae junctions [113, 114]. It is clear that the fragmentation of the mitochondria during apoptosis is independent of caspase activity [115], and it takes place through two coordinated, but independent, events: opening of cristae junctions, where cytochrome *c* is bound, and formation of the outer membrane pores [87, 111, 116–120].

DRP-1 colocalizes with the BAX/BAK pores [107, 121, 122] where it promotes disintegration of the mitochondrial network. The fragmented mitochondria collapse in a perinuclear pattern and show decreased and non-directed motility. Consistent with the increased mitochondrial fragmentation, mitochondrial fusion has also been shown to be blocked once apoptosis is activated [123].

Endoplasmic reticulum (ER) tubules frequently cross paths with mitochondria at points of impending fission and mark sites of mitochondrial division, a phenomenon known as ER-associated mitochondrial division (ERMD) [75, 124]. These studies also indicate that the ER might play an active role during the early stages of fission, even before DRP-1 severs the mitochondria. At these “hotspots” mitochondria are constricted and allow for assembly of the diversion DRP helix. The ER may be able to alter mitochondrial membrane composition, facilitate factors, such as MFF, on the inside and/or outside of mitochondria to promote fission; however, the mechanisms underlying ER-mitochondrial microdomain or ER-mitochondrial constriction is not well established [75]. While the function of DRP1 in constricting the mitochondria has been established using cancer cell lines, the exact mechanism by which DRP-1 regulates fragmentation during apoptosis or by which it maintains the constitutive fragmentation of the mitochondrial network in stem cells and some cancer stem cells is less clear. It will be interesting to determine if the DRP-1 mechanism of action and function in homeostatic conditions and stressed conditions is modified in stem cells and cancer stem cells where mitochondria are maintained in a more fragmented state.

While the high mitochondrial priming described in the first section and the increased mitochondrial fragmentation are two fundamental features that accompany entry into the pluripotent state, the protein network and exact signaling events that are modulating these changes remain elusive. We speculate that the BCL-2 family mediates both of these properties, and that reprogramming of differentiated cells into stem cells provides a useful tool to dissect this intriguing possibility. Examining the connection between mitochondrial priming, fragmentation and the acquisition of a particular stem cell fate opens an exciting opportunity for future studies to determine whether these properties are mechanistically related.

## DECREASED MITOCHONDRIAL-DEPENDENT METABOLISM

The increased sensitivity to cell death as well as the structural and functional remodeling of the mitochondrial network described in previous sections are accompanied by an essential switch from oxidative phosphorylation (OXPHOS) to glycolysis during reprogramming. The detailed molecular mechanisms and the temporal regulation underlying this switch remains unclear. ESCs and iPSCs have a similar metabolic profile to many cancer cells that predominantly depend on glycolysis for rapid proliferation and self-renewal. There are landmark papers on the characterization of the differences in metabolic profiles between various stages of pluripotency in mPSCs (i.e., naïve PSCs, primed PSCs and epiblast stem cells) [125–127]. In this section we will describe the main metabolic pathways involved in promoting cellular reprogramming and maintaining pluripotency in human PSCs [128].

### Oxidative phosphorylation (OXPHOS)

OXPHOS is a primary source of ATP production in eukaryotic cells. A series of enzymes residing in the inner mitochondrial membrane oxidize the products of glycolysis and citric acid cycle to release protons into the inter-membrane space in the presence of oxygen. This establishes a proton gradient that drives the ATP synthase which shuttles back the hydrogen ions and produces 30 ATP molecules in the process, but it requires oxygen.

Energy production of PSCs rely heavily on glycolysis over OXPHOS [129–131]. While OXPHOS results in increased production reactive oxygen species (ROS) that could be deleterious, low ROS levels produced at the mitochondria have been shown to be critical for signaling and cell survival [132]. Thus, maintaining functional respiratory complexes may be critical for generating endogenous levels of ROS critical for stem cell renewal and pluripotency. Mitochondrial uncoupling protein 2 (UCP2)-mediated suppression of OXPHOS is required for the maintenance of pluripotency. UCP2 decouples glycolysis from OXPHOS by shunting pyruvate out of the mitochondria [133]. However, it is not completely understood whether this suppression of OXPHOS in PSCs still results in permissive low levels of endogenous ROS. Redox levels and pathways involved in redox signaling have not been thoroughly studied during cellular reprogramming, and remain an open area of investigation.

### Glycolysis

Glycolysis involves ten reactions in the cytosol that rapidly catabolizes each six-carbon glucose molecule to produce two ATP molecules, without the requirement of oxygen. Glycolysis generates pyruvate, which in most cell types, can be shunted into two metabolic fates: in the presence of oxygen, pyruvate enters the mitochondria where it is oxidized to acetyl-CoA via pyruvate dehydrogenase (PDH), whereas in the absence of oxygen, pyruvate is reduced into lactate via lactate dehydrogenase (LDH) [134–138]. The intermediates in glycolysis can also be shunted into macromolecule synthesis during rapid cell growth. Thus, while it is a less efficient source of energy, glycolysis can generate both anabolic growth intermediates and ATP very rapidly owing to the much higher speed of glycolysis reactions. Primed pluripotent stem cells utilize glycolysis as a primary source of energy, converting glucose to lactate instead of directing the produced acetyl-CoA into the tricarboxylic acid cycle (TCA) cycle at the mitochondria as seen in mature differentiated cells which mostly rely on the TCA cycle for energy production [139–141]. Glycolysis in PSCs is crucial for maintaining pluripotency. In fact, conversion to glycolysis is necessary for successful reprogramming as inhibition of glycolysis in hESCs has been shown to result in apoptosis and cell cycle arrest [142].

Interestingly, hypoxic conditions are known to stimulate glycolysis and have been shown to prevent the spontaneous differentiation of hESCs [143, 144]. As hypoxic niches are the primary residing locations for PSCs, glycolytic metabolism might represent an adaptation to their surroundings. The increased senescence in MSCs cultured under normoxia (21% O_2_) compared to when cultured under 5% O_2_ levels supports this hypothesis [145]. Hypoxia inducible factor-1 (HIF-1) regulates transcriptional activities and induces a switch from OXPHOS to glycolysis followed by suppression of mitochondrial biogenesis in response to hypoxia and thus, represent an important link between mitochondrial metabolism in PSCs and cellular reprogramming [146–149].

### Methionine metabolism

Human PSCs use high levels of methionine instead of threonine as seen in murine PSCs [126, 150]. This is because threonine dehydrogenase, which catabolizes threonine into 2-amino-3-ketobutyrate, evolved into a pseudogene in humans, unlike most other mammals [151]. Methionine provides the methyl group for many histone methyltransferases. Uptake of methionine from the culture media is required to maintain S-adenosylmethionine (SAM) levels in hPSCs. SAM is a methyl donor for histone methyltransferases (HMT) and DNA methyltransferases (DNMTs). Methionine deprivation results in a rapid decrease in SAM, loss of H3K4me3, and reduced NANOG expression which triggers human PSCs to differentiate. Prolonged methionine deprivation leads to cell cycle arrest and apoptosis in human PSCs [150]. SAM is consumed by NNMT (nicotinamide N-methyltransferase) which has been found upregulated in naïve hPSCs [152]. Thus, appropriate levels of methionine in the culture media are required to maintain SAM levels and global DNA and histone methylation, which are important for the maintenance of pluripotency [126].

Recent findings by Vernardis and collaborators correlate changes in metabolism to the differential responses of hiPSCs and hESCS, after prolonged exposure to ROCK (Rho kinase) inhibitor [153]. ROCK inhibition enables maintenance of stem cell phenotype. The authors assessed the effect of ROCK inhibition on the metabolic characteristics of cells over a 96 hours (h) of culture period. Both hPSCs and hiPSCs showed downregulation of the methionine pathway following 12 h and 24 h exposure and upregulation after 48 h. This suggests that both hPSCs and hiPSCs lose their highly proliferative characteristics after exposure to ROCK inhibition and regain their proliferation potential following adaptation to the new culture conditions. Interestingly, though, no differences in the gene expression, protein levels and physiology of hESCs and hiPSCs were observed, a differential expression of metabolic regulators p53 and mTORC1 revealed the fluctuating state of metabolism. Collectively, these and other studies have revealed the importance of methionine metabolism in integrating extrinsic amino acid information with the intrinsic epigenomics and pluripotency state to determine the cell fate of PSCs [154–157].

### Acetyl-CoA

While the importance of metabolism on SAM levels and global methylation patterns has been studied in detail, the regulation of other key metabolites, such as acetyl-CoA, in PSCs and iPSCs needs additional investigation. Acetyl-CoA is not only a substrate for TCA cycle but it may serve for histone acetylation in PSCs for maintaining their pluripotency [158]. By performing a high resolution nuclear magnetic resonance experiment, Moussaieff and collaborators identified a metabolic transition marked by loss of acetyl-CoA that leads to histone deacetylation, during hPSC differentiation [159]. The lipogenic enzyme acetyl-CoA carboxylase has been found upregulated in iPSCs and their inhibition is linked to decreased reprogramming efficiency [160]. Likewise, chemical inhibition of histone deacetylases is shown to promote reprogramming of somatic cells to iPSCs [161]. These studies suggest that it is via shunting of pyruvate-cytosolic acetyl-CoA that glycolysis may contribute to pluripotency regulation [162].

A recent study reported that cytosolic acetyl-CoA, whichis produced through glycolysis and the pyruvate-derived citrate flux via ATP citrate lyase (ACLY) is inhibited during PSC differentiation [159]. Acetyl-CoA blocks histone deacetylation and stem cell differentiation, while acetate, an alternative precursor of cytosolic acetyl-CoA, delays PSC differentiation by promoting histone acetylation in a dose-dependent fashion [159].Thus, a glycolytic switch that regulates histone acetylation can have a profound effect on the ability of stem cells to differentiate [159]. Of note, the decrease in OXPHOS might be causal for the activation of glycolysis during reprogramming, as (OXPHOS-derived) ATP is a potent allosteric inhibitor of a number of enzymes involved in glycolysis [163]. In sum, glycolysis may remodel the metabolome and facilitate the reprogramming of somatic cells into iPSCs [129, 164, 165].

While it is conventionally thought that metabolism is altered as a consequence of the chosen cell fate (i.e., metabolic demand drives the cell's metabolic program), an intriguing possibility is that metabolism itself can dictate stem cell renewal and/or differentiation by altering transcriptional networks that modulate cell fate [134]. It will be interesting to determine when during reprogramming and differentiation these metabolic changes take place. Characterization of the temporal regulation of metabolism, mitochondrial dynamics, and apoptosis during stem cell fate decisions could provide critical insight into if the changes in these processes are a result of the stem cell state, or a necessary factor for maintenance of the state of the cell.

## CONCLUDING REMARKS

Mitochondria are a crucial source for fuel and intermediate metabolites that are essential for many cellular functions. These organelles house key proteins involved in the regulation of apoptosis and metabolism while maintaining a highly dynamic network that is essential for their function. Decades of studying these organelles in isolation have allowed for the elucidation of the main components involved in apoptosis, OXPHOS and mitochondrial dynamics, but we know less about the crosstalk between these pathways and how they are regulated as mitochondria communicate within themselves and with other cellular membranes. Many questions about mechanisms and integration of these mitochondrial changes and signaling networks remain unanswered. Future studies should be aimed to understand how these pathways intersect to regulate cell survival, what factors control the switch in mitochondrial network status, turnover, and connectivity to the ER as cells undergo reprogramming; how do changes in metabolic state and metabolite levels affect epigenetic enzymes that regulate gene expression; and how is respiratory capacity increased with differentiation and lowered with reprogramming. Answering these questions could lead to a complete understanding of how mitochondrial biology and function modulate cell fate.

Recent advances in the field of stem cell biology and in cellular reprogramming technology in particular, have created new opportunities in understanding human disease, drug discovery, and regenerative medicine. The generation of iPSCs reveals the remarkable plasticity associated with differentiated cells and provides unprecedented opportunity to model diseases using patient samples. In addition to transcriptional and epigenetic remodeling, cellular reprogramming is also accompanied by dramatic changes in the structure and function of the mitochondria. In this review, we describe three main mitochondrial events linked with reprogramming: increase of cell death sensitivity, fission of mitochondrial network, and decreased OXPHOS. We discussed potential mechanisms underlying the rewiring of the cellular state. It is clear, that we are just beginning to understand the integral roles played by mitochondrial morphology and function in development and diseases. There are also other areas of exciting opportunities. Today, it is possible to culture additional types of human pluripotent stem cells that model cells from preimplantation and post-implantation embryos [125, 166]. These different phases of pluripotency, for example, naïve state (which resembles the pre-implantation blastocyst inner cell mass), primed state (which resemble the post-implantation epiblast cells) and extended PSCs (which can generate embryonic and extraembryonic tissues), can all now be cultured *in vitro* simply by adapting different cell culture parameters. This possibility opens the potential to examine the function of mitochondrial morphology, dynamics and function in the context of the developmental potential of all of these model systems.

Pioneer work from Ruth Slack's group [11] highlighted the physiological significance of mitochondrial dynamics for stem cell identity and fate during mouse brain development. Studies in human brain have been limited by the lack of model systems. The emergence of three-dimensional models, known as organoids [167–170], has opened a new avenue of discovery and could provide an additional model system to study the effects of disrupting mitochondrial morphology/function on early human brain development. While human stem cells provide many advantages, it is difficult to model many stages of maturation, such as neurodevelopmental processes of myelination, gliogenesis etc., which may not occur in the time that a monolayer culture can be maintained. Understanding the molecular events governing the mitochondria-related hallmarks described in this review, in the context of three-dimensional tissues could reveal new areas of discovery on the impact of disrupting mitochondrial function in the development of degenerative diseases. Organoids better recapitulate functional, structural, and architectural complexity of organs and their cellular diversity and thus, provide a powerful tool to dissect the principles of mitochondrial biology we have learned from other systems.

Recent discoveries have evidenced the interplay between mitochondrial dynamics, intermediate metabolism and epigenetics, thus, the traditional views of mitochondria as isolated organelles and of metabolism as a developmental byproduct have largely been refuted. To what extent mitochondrial morphology and cellular metabolism are directly wired to cellular transitions associated with development and reprograming warrants future investigations. Recent studies shed light into the essential requirement for a fine-tuned regulation of mitochondrial morphology and function for the maintenance of stem cell properties. New studies in these areas could have farreaching implications facilitating innovations leading to new treatments for patients affected by neurodevelopmental and neuropsychiatric disorders.

## Abbreviatons

BAK: BCL-2 antagonist killer
BAX: BCL-2 associated X
BCL-2: B-Cell Lymphoma-2
BID: BCL-2 interacting domain death agonist
DRP: dynamin-related GTPase protein
ER: endoplasmic reticulum
ESC: embryonic stem cell
hPSC: human PSC
iPSC: induced PSC
OXPHOS: oxidative phosphorylation
PSC: pluripotent stem cell
ROCK: Rho kinase
ROS: reactive oxygen species
SAM: S-adenosylmethionine
TCA: tricarboxylic acid
